# Identifying Sleep Disorders From Search Engine Activity: Combining User-Generated Data With a Clinically Validated Questionnaire

**DOI:** 10.2196/41288

**Published:** 2022-11-23

**Authors:** Mairav Cohen Zion, Iddo Gescheit, Nir Levy, Elad Yom-Tov

**Affiliations:** 1 Dayzz Herzeliya Israel; 2 Microsoft Research Herzeliya Israel

**Keywords:** sleep disorders, search engine queries, search advertising, internet, Bing, sleep, machine learning, questionnaire

## Abstract

**Background:**

Sleep disorders are experienced by up to 40% of the population but their diagnosis is often delayed by the availability of specialists.

**Objective:**

We propose the use of search engine activity in conjunction with a validated web-based sleep questionnaire to facilitate wide-scale screening of prevalent sleep disorders.

**Methods:**

Search advertisements offering a web-based sleep disorder screening questionnaire were shown on the Bing search engine to individuals who indicated an interest in sleep disorders. People who clicked on the advertisements and completed the sleep questionnaire were identified as being at risk for 1 of 4 common sleep disorders. A machine learning algorithm was applied to previous search engine queries to predict their suspected sleep disorder, as identified by the questionnaire.

**Results:**

A total of 397 users consented to participate in the study and completed the questionnaire. Of them, 132 had sufficient past query data for analysis. Our findings show that diurnal patterns of people with sleep disorders were shifted by 2-3 hours compared to those of the controls. Past query activity was predictive of sleep disorders, approaching an area under the receiver operating characteristic curve of 0.62-0.69, depending on the sleep disorder.

**Conclusions:**

Targeted advertisements can be used as an initial screening tool for people with sleep disorders. However, search engine data are seemingly insufficient as a sole method for screening. Nevertheless, we believe that evaluable web-based information, easily collected and processed with little effort on part of the physician and with low burden on the individual, can assist in the diagnostic process and possibly drive people to seek sleep assessment and diagnosis earlier than they currently do.

## Introduction

Studies have suggested that nearly all adults will experience sleep disturbances over the course of their lives, and that up to 40% of the population experiences chronic sleep disorders [1]. Common sleep disorders include insomnia, sleep apnea, circadian rhythm disturbances, and chronic insufficient sleep. These sleep disorders cause a major health and societal burden, contributing to a loss of personal well-being, development of chronic disease, loss of work productivity and performance, and spikes in accidents that result in needless injury and death.

Sleep disorders are diagnosed by specialized physicians, limiting access and increasing the time to diagnosis in people with sleep disorders [2]. The shortage of trained sleep experts and the lack of geographic accessibility, combined with a convoluted sleep care pathway have led to long waitlists for sleep diagnostics and intervention, with many patients dropping off along the care pathway.

Sleep disorders can be diagnosed using a variety of techniques, using different sources of subjective and objective information and with different levels of invasiveness. Some disorders, such as insomnia, can typically be initially assessed using periodic screeners by health care professionals to identify potential symptoms, while an in-depth sleep history is essential for diagnosis and treatment matching. For example, sleep apnea requires overnight sleep testing (in the laboratory or at home). Other conditions may require in-laboratory daytime sleep testing, at-home continuous sleep-wake monitoring (typically with medical-grade, wrist-worn activity monitors), physical and neurological assessments, and even blood analysis [3]. Interestingly, wearable devices for sleep monitoring have shown limited reliability [4], though some researchers have reported success in predicting sleep quality [5,6].

One approach for initial screening of sleep disorders is through web-based questionnaires. Dayzz is a digital sleep program that provides comprehensive web-based care for multiple sleep conditions. As part of the program, the Digital Sleep Questionnaire (DSQ) [7] is administered. The DSQ is a brief, clinically validated, questionnaire that, using machine learning algorithms, can provide an assessment of 4 common sleep disorders.

People’s web-based activity, especially their interactions with search engines, have been shown to enable early screening for a range of medical conditions including diabetes [8], Parkinson disease [9], stroke [10], and several types of cancer [11,12]. The reason for the ability to screen using web-based interactions include the ubiquity of these services in people’s daily lives and their service as a gateway to information (especially health information) for the vast majority of the population [13]. Interactions with search engines are especially useful in this regard because they are plentiful and are perceived as sufficiently anonymous, allowing people to express their truthful information needs, without fear that their sensitive or personal information will be divulged to third parties [14]. We note that social media platforms such as Twitter have also been used to characterize sleep issues [15] but not sleep disorders.

In this study, we aim to evaluate whether search engine activity can be used to screen for possible sleep disorders. We show that a machine learning model based on search engine activity can predict the risk for specific sleep disorders, although currently, the quality of the prediction is insufficient as a sole method for screening. Nevertheless, we believe that evaluable web-based information, easily collected and processed with little effort on part of the physician and with low burden on the individual, can assist in the diagnostic process and possibly drive people to seek diagnosis earlier than they currently do.

## Methods

### Overview

We implemented a novel experimental protocol whereby participants are recruited through internet-based advertisements after they indicated their interest in sleep disorders. People who clicked on the advertisements and consented to participate in the study were referred to a digital sleep questionnaire and were promised general feedback on their sleep patterns as an incentive to complete the questionnaire. They were then asked for permission to contribute their search engine data to the study. Finally, we linked the outcome of the sleep questionnaire with search engine data of the people who consented. These data are used to predict the questionnaire outcome.

### Recruitment

We advertised on Microsoft Advertising to US-based users of Bing who queried for one of a range of sleep disorder–related terms (see Multimedia Appendix 1); those people were shown 1 of 12 advertisements, which are listed in Multimedia Appendix 2. People who clicked on the advertisements were referred to a dedicated landing page.

The web page described the experiment to potential participants. Those who consented to join were administered the DSQ [7]—a brief, clinically validated questionnaire that, using machine learning algorithms, can provide an assessment of 4 common sleep disorders: risk for obstructive sleep apnea (OSA), delayed sleep phase syndrome (DSPS), insomnia, and insufficient sleep syndrome (ISS). They were then provided general feedback on their sleep and asked if they agree to have their Bing search data linked to the results of the DSQ and analyzed.

The data of those users who consented were extracted from up to 1 year prior to the date of questionnaire completion and up to the date of the questionnaire. The data comprised an anonymized user identifier, the time and date of the query, the zip code of their location, and indicators calculated from user interactions with search results’ pages, including the duration of the search session, the number of links clicked, the time they spent on each link, whether automatic spelling correction was used, and whether the user scrolled down the search page.

The campaign was gradually improved over time by providing a conversion optimization signal to the advertising campaign, such that completion of the DSQ was considered a conversion “worth” US $1 and the same combined with downloading of a dedicated sleep improvement app was set to US $10.

### Analysis

We represented the activity of users through a set of aggregate attributes detailed in Textbox 1. These attributes represent several factors that could typify a sleep disorder or are risk factors for it. Specifically, we quantified diurnal activity profiles, as evident from query use; measures of interaction with the search engine (eg, time to first click), which are proxies for cognitive function; and keywords, which reflect risk factors, behaviors that pertain to sleep and its disorders, and the sleep disorders themselves. A list of the latter was developed on the basis of the literature.

Since there is no simple definition of when a day begins and ends for people with sleep disorders, we chose the hour with the lowest level of activity among participating users to be the start of a day (ie, a 24-hour period). Thus, 2 AM was set as the start of the day.

To facilitate comparison of users in our data set with the general population, we extracted a random sample of US-based Bing users who were active on the same dates as the study participants and presented the percentage of queries at each hour of the day made by these users. We refer to these users as the “control population.”

Each user was labeled in accordance with the DSQ as at risk for up to 2 of 4 sleep disorders (OSA, DSPS, insomnia, and ISS). We attempted to predict each of the sleep disorders using a binary classifier. The binary classifier used was a random forest with 100 trees.

The performance of the models was evaluated using leave-one-out cross-validation. The best attribute groups were selected using sequential forward feature selection on the training set.

Only users with at least 14 days of data and 50 queries were retained for analysis.

**List of attributes for predicting sleep disorders.**

**Query time:**

- Percentage of queries at each hour of the day
- Time between consecutive queries (average, 5th and 95th percentiles)
- Average first and last hour of queries on each day
- Whether the user had noncontiguous activity during night hours
- The difference between the hourly activity rate on weekdays compared to that on weekends
- Fraction of queries prior to sunrise
- Fraction of queries after sunset

**Activity attributes:**

- Average click count on each results page
- Average time on links on each results page
- Average session duration
- Average scrolled distance on each results page
- Average time to first click on each results page
- Average time to last click on each results page
- Average, minimum, maximum, and median of the query text probability
- Average number of sessions per day
- Average number of queries that the user repeated
- Average number of queries which mentioned a medical symptom
- Average number of queries that mentioned a medical diagnosis
- Average number of queries that mentioned a medical drug

**Terms:**

- Number of times a user queried for the following specific terms (terms were used separately, but are grouped here for convenience):
  Apnea, insomnia
  Snoring, gasping, headache, sleepiness, waking, snoozing, dozing, awake, alert, appetite, depression, anxiety, diabetes, obesity, weight loss, memory, oxygen, breath, tired, exhausted, fatigue, no energy, nap, hypertension, high blood pressure, HBP, emotion, and mood
  Jet lag, porn-related terms, accident, mistake, coffee, caffeine, energy drink, alcohol, alcoholic drinks, smoking, nicotine, baby-related terms, attention, concentration, pregnancy, movie, TV, credit union, loan, cannabis, CBD, THC, marijuana, gambling-related terms, online banking, inbox, CNN, and zoom
  Sleeping pill, hypnotic, sedative, sleep aid, melatonin, and exercise

**Query topics:**

- The distribution of the users’ query topics into 60 topics according to a proprietary classifier.

**Demographics:**

- User age
- User sex

### Ethical Considerations

The methods were performed in accordance with relevant guidelines and regulations and approved by the institutional review board of Advarra Inc (Pro00045152).

## Results

We recruited participants between November 11, 2020, and May 19, 2021. Campaign statistics are provided in Multimedia Appendix 2. A total of 582 users clicked on the advertisements and were referred to the DSQ web page. Of them, 397 consented and completed the DSQ. Among users who completed the DSQ, 305 were matched to their Bing data and 132 had at least 14 days of data and 50 queries.

**Figure 1:**
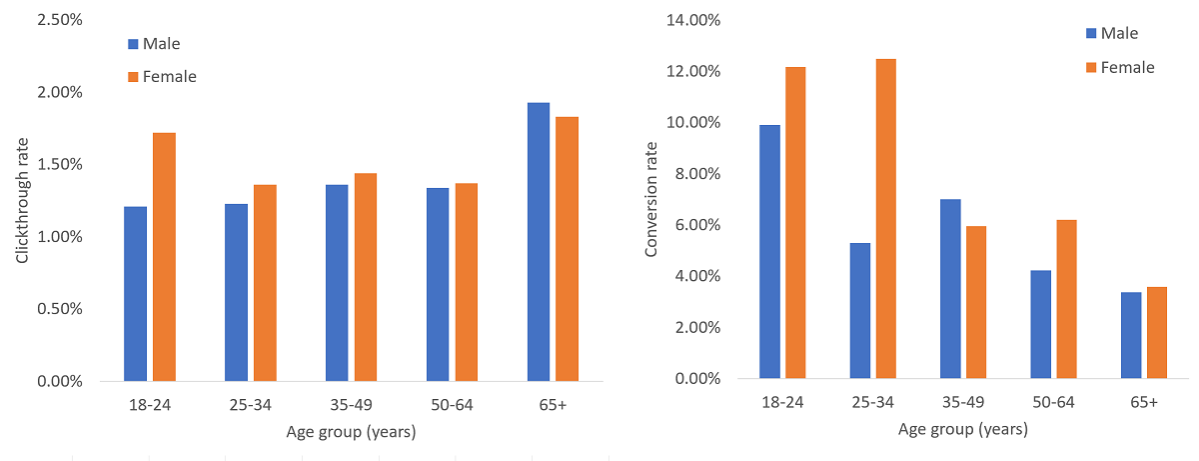
Clickthrough rate (left) and conversion rate (right) by age and sex. Blue bars denote males and orange bars denote females.

The age and sex distribution, as provided by the advertising system, is shown in Figure 1. Two measures are provided: the percentage of advertisements clicked on among all those shown (referred to as the clickthrough rate [CTR]), and the percentage of questionnaires completed among all those who clicked on the advertisements (denoted by conversion rate). As shown in Figure 1, younger females were more likely to respond to the advertisements by clicking on them (higher CTR) and to complete the DSQ (higher conversion rate). Younger people were overall more likely to complete the DSQ (that is, to “convert”).

**Figure 2:**
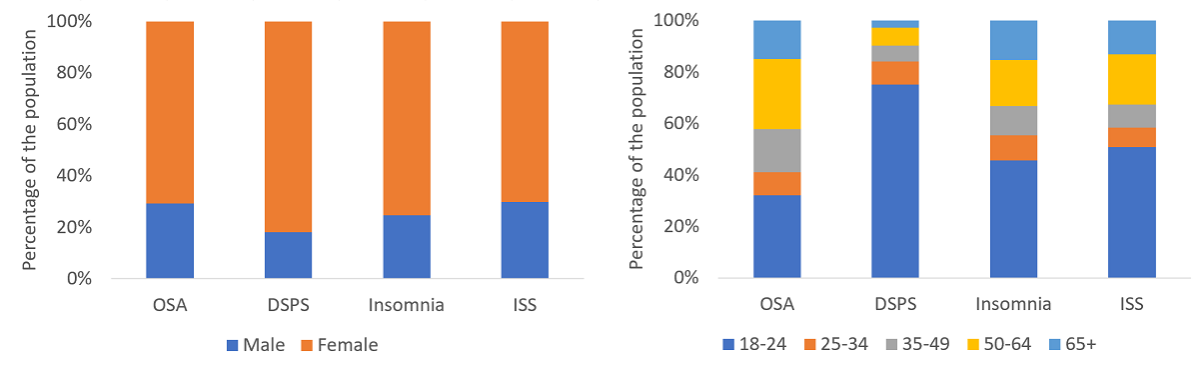
Distribution of ages (left) and sex (right) among sleep disorders. DSPS: delayed sleep phase syndrome; ISS: insufficient sleep syndrome; OSA: obstructive sleep apnea.

According to the DSQ, 58 participants were at risk for OSA, 52 for DSPS, 89 for Insomnia, and 46 ISS. The distribution of ages and sex among sleep disorders is shown in Figure 2. When comparing among the conditions, males accounted for a larger proportion of individuals at risk for ISS and those who are at risk of OSA, while those at risk for DSPS accounted for the largest proportion of females. DSPS was most prevalent among young participants; risk for OSA, in older participants.

**Figure 3:**
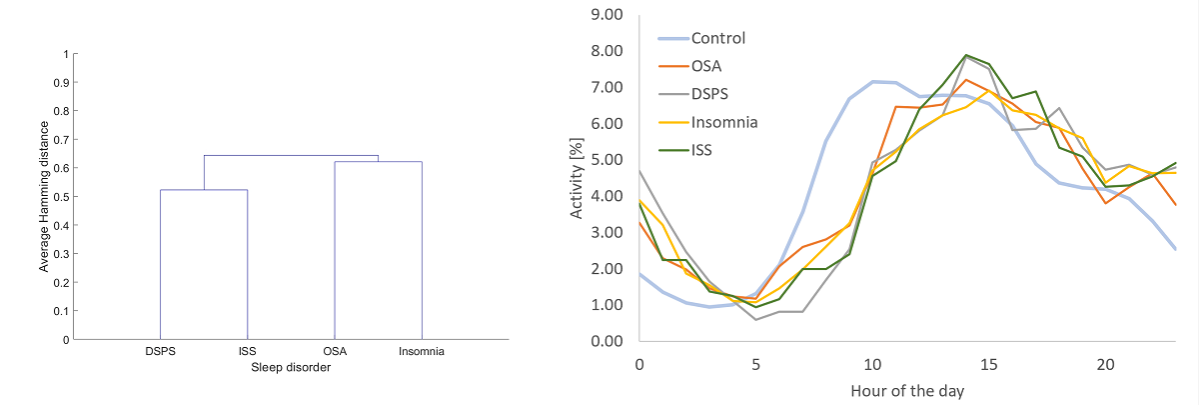
Comorbidity similarity among sleep disorders according to the users who shared them (left) and the daily activity profile of users (right). The distance among conditions was computed using Hamming distance of users. DSPS: delayed sleep phase syndrome; ISS: insufficient sleep syndrome; OSA: obstructive sleep apnea.

We estimated the comorbidity similarity between sleep disorders by the Hamming distance applied to the matrix of participants and their conditions. Figure 3 shows the comorbidity similarity among conditions and the activity profile of users over the day. As shown in Figure 3, people with DSPS were most likely to also have ISS, and those at risk for OSA were most likely to also have insomnia. The daily activity profile of users is similar among sleep disorders (though most similar among users who were at the risk of OSA and those with ISS), but very different from that of the control population. Specifically, the control population begin their activity earlier in the morning hours and end their activity earlier at night, compared to those with sleep disorders. The best correlation between the activity of the control population and the activity of all users with sleep disorders was approached when the activity of the former is shifted by 2 hours for the risk of OSA and insomnia, and by 3 hours for DSPS and ISS. The improvement in correlation due to these time shifts was significantly different (*P*<.01 with Bonferroni correction).

**Figure 4:**
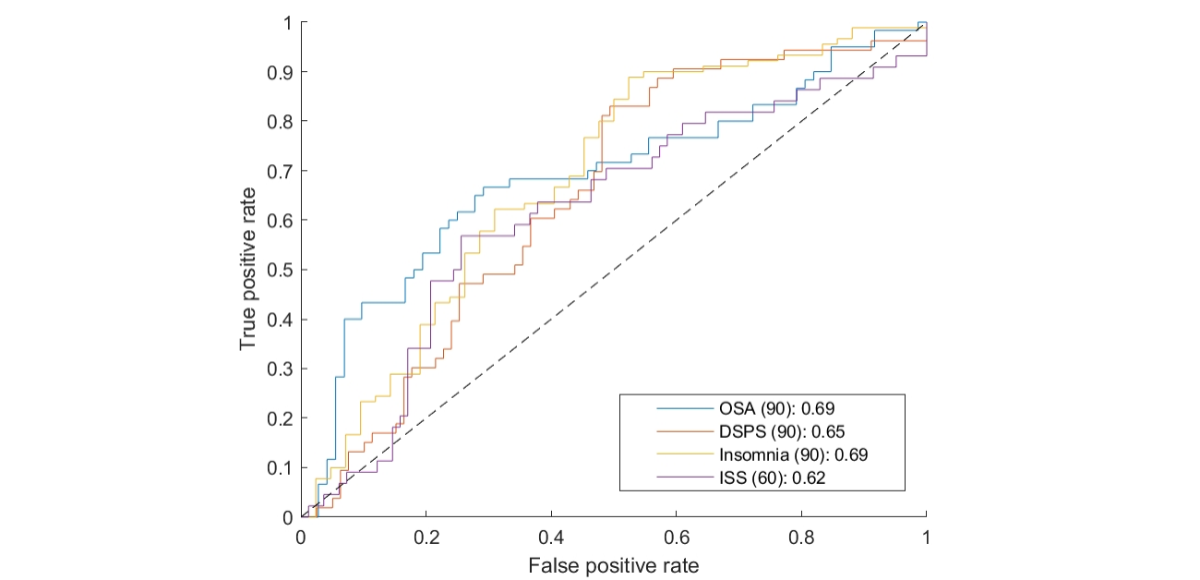
Receiver operating characteristic curve for the 4 sleep disorders. The numbers in the legend indicate the number of days during which data were obtained for each condition and the area under the curve. DSPS: delayed sleep phase syndrome; ISS: insufficient sleep syndrome; OSA: obstructive sleep apnea.

Table 1 shows the performance of the sleep disorder classifiers as evaluated by their area under the curve, together with attribute classes most commonly selected for classification and the number of days of data that approached the best performance. For the latter, we experimented with using different values between 15 and 120 days. Figure 4 shows the receiver operating characteristic curve for each sleep disorder.

We also evaluated which terms would best predict each of the sleep disorders if used without any other attribute classes. This was achieved by finding the terms that increase in prediction error if the values of that term are permuted for the test observations, using the appropriate MATLAB function. The results are summarized in Textbox 2.

**Table 1:** Classifier area under the curve, the best number of days selected for use by the classifier from prior to the Digital Sleep Questionnaire and until the day of the questionnaire, and the most commonly selected attributes.

Sleep disorder classifier	Area under the curve	Number of days	Selected attribute classes
Risk for obstructive sleep apnea	0.69	90	Query time, activity attributes, and demographics
Delayed sleep phase syndrome	0.65	90	Query time and demographics
Insomnia	0.69	90	Activity attributes, query topics, and demographics
Insufficient sleep syndrome	0.62	60	Query time, activity attributes, and specific terms

**Most predictive terms of each sleep disorder.**

**Risk for obstructive sleep apnea:**

- Coffee
- Tired, exhausted, fatigued
- Waking
- Hypertension
- Awake

**Delayed sleep phase syndrome:**

- Headache
- Awake
- Porn-related keywords
- Smoking-related keywords
- Pregnancy-related keywords

**Insomnia:**

- Coffee
- Online banking, inbox, CNN, zoom
- Sleepiness
- Waking
- Awake

**Insufficient sleep syndrome:**

- Exercise
- Depression
- Memory
- Anxiety
- Pregnancy-related keywords

## Discussion

Sleep disorders have a significant personal and societal cost. However, barriers to wide-scale screening and diagnosis prevent timely treatment for many people with such disorders. There is a need for more accessible and affordable tools that enable remote screening and diagnosis of sleep disorders, especially those which can be performed passively without any effort from the user. Here we test whether interactions with search engines could be used to identify persons at risk for sleep disorders, as a first step in navigating them to further evaluation and intervention.

Our campaign was used to the greatest extent by younger females. In general, younger people were more likely to complete the web-based screening questionnaire. This is in agreement with past research, which showed that younger people are more likely to interact with web-based medical questionnaires [11,16]. However, this also underscores a potential bias in screening using an advertisements-based questionnaire.

The comorbidity among certain sleep disorders found in our sample is in line with that reported in previous studies, showing that risk for OSA and insomnia often coexist [17], and that unmanaged DSPS can often lead to chronic insufficient sleep [18]. Moreover, we found that adults with sleep concerns and potentially increased unwanted or unregulated wakefulness at night tend to browse differently than those without sleep concerns, with search patterns that started and ended later in the day. Perhaps unsurprisingly, we also found that those with natural delays in their biological clock, a distinguishing characteristic of DSPS, were even more shifted in these internet usage patterns than those at risk for other sleep disorders. These findings suggest that when developing algorithms to identify persons at the risk of sleep disorders—in addition to specific sleep—and related health and behavior search content that we found to support differing sleep disorder risk, metadata may offer more fine-grained activity patterns, increasing the predictive performance of the models. Interestingly, independent of the other predictor attribute classes, the search terms that participants used were indicative of their underlying risk for certain sleep disorders; in many cases, the search terms they chose were diagnostic symptoms, functional deficits, or common comorbidities of the specific sleep disorder that they were at the risk of; for example, hypertension and sleepiness-related terms in the case of risk for OSA [19], and mental health and cognitive deficits in the case of chronic insufficient sleep [20]. Our findings are promising; however, future research should focus on additional content, query parameters, and time periods that can improve and refine the predictive ability of search engine data to identify individuals at the risk of sleep disorders.

The area under the curve for all 4 conditions ranged between 0.62 and 0.69, indicating that search engine data are predictive of sleep disorders, with the prediction being of medium accuracy. We note that sleep disorders are known to be a difficult problem to assess, as evident, for example, in the low agreement among experts at different laboratories on the correct interpretation of diagnostic tests, such as polysomnography, in people with sleep disorders [21], and modest agreement rates between telemedicine and in-person diagnosis [22]. Moreover, our data show that the last 60 to 90 days of search data reach the highest prediction quality. This may be because people responded more readily to the campaign advertisements when their symptoms were more acute.

Our study has several limitations. First, our recruitment occurred among people who were interested or concerned about their sleep. Thus, our results focus on the ability to inform people who are already concerned about possible sleep problems and not the ones who are not part of the general population. Among people who saw our advertisements, younger females were more likely to respond to the advertisements. Among those who clicked on the advertisements, younger people were more likely to complete the DSQ. Thus, our prediction model is biased toward younger people. Future work will attempt to distinguish between healthy populations and those with sleep disorders as well as obtain a more balanced sample of people with sleep disorders. The former will allow a wider application of the model to additional populations, while the latter will ensure that the prediction is applicable to people of all ages.

## Appendix

Multimedia Appendix 1 List of campaign keywords.

Multimedia Appendix 2 Campaign statistics.

## Abbreviations

CTR: clickthrough rate
DSPS: delayed sleep phase syndrome
DSQ: Digital Sleep Questionnaire
ISS: insufficient sleep syndrome
OSA: obstructive sleep apnea
