# Supplementary material for: Identifying Sleep Disorders From Search Engine Activity: Combining User-Generated Data With a Clinically Validated Questionnaire
Source: J Med Internet Res. 2022 Nov 23;24(11):e41288. doi: 10.2196/41288 (PMC9730212; doi:10.2196/41288)
Supplement: Multimedia Appendix 1 [file jmir_v24i11e41288_app1.docx]

## Appendix A: List of campaign keywords

Note that each mention of “sleep disorder” in the list below was changed to each of the 4 sleep disorders examined in this study.

suspected sleep apnea, treatment options for suspected sleep apneas, how to cure suspected sleep apneas, how to cure suspected sleep apneas, how to treat suspected sleep apneas, suspected sleep apnea treatment, do i have a suspected sleep apnea, Do I have a suspected sleep apnea, suspected sleep apnea home test , Signs of suspected sleep apnea , Test for suspected sleep apnea, Symptoms of suspected sleep apnea, insufficient sleep syndrome, treatment options for insufficient sleep syndromes, how to cure insufficient sleep syndromes, how to cure insufficient sleep syndromes, how to treat insufficient sleep syndromes, insufficient sleep syndrome treatment, do i have a insufficient sleep syndrome, Do I have a insufficient sleep syndrome, insufficient sleep syndrome home test , Signs of insufficient sleep syndrome , Test for insufficient sleep syndrome, Symptoms of insufficient sleep syndrome, delayed sleep phase syndrome, treatment options for delayed sleep phase syndromes, how to cure delayed sleep phase syndromes, how to cure delayed sleep phase syndromes, how to treat delayed sleep phase syndromes, delayed sleep phase syndrome treatment, do i have a delayed sleep phase syndrome, Do I have a delayed sleep phase syndrome, delayed sleep phase syndrome home test , Signs of delayed sleep phase syndrome , Test for delayed sleep phase syndrome, Symptoms of delayed sleep phase syndrome, treatment options for insomnias, how to cure insomnias, how to cure insomnias, how to treat insomnias, insomnia treatment, do i have a insomnia, Do I have a insomnia, insomnia home test , Signs of insomnia , Test for insomnia, Symptoms of insomnia, treatment options for sleep disorders, how to cure sleep disorders, how to cure sleep disorders, how to treat sleep disorders, sleep disorder treatment, sleep doctors, Prescription medications for sleep , Sleeping pills, OTC Sleep aids, Natural Sleep aids, Can't wake up in the morning, Unable to stay awake, Morning headaches, Dry mouth in the morning, Gasping during during sleep, Pauses in breathing during sleep, Gasping during the night, Pauses in breathing at night, Choking during sleep, Choking at night, Snoring, how to get good sleep naturally, how do i sleep better, how to fall asleep better, how to improve sleep habits, why can i not sleep at night, how to improve your sleep, what causes restless sleep, what can i do to help me sleep, how to get quality sleep, do i have a sleep disorder, what to do to fall asleep, how to sleep better at night, how to sleep properly, how to get more sleep, how to improve sleep, how to sleep through the night, how to get better sleep, how many hours of sleep do i need, how to sleep better, how much sleep do i need, why can't i sleep, how to fall asleep fast, reasons for not falling asleep, having difficulty sleeping, anxiety and sleep problems, trouble getting to sleep, sleeping problems causes, causes of daytime sleepiness, have trouble sleeping, anxiety sleep problems, problems falling asleep, falling asleep during the day, difficulty falling asleep, feeling sleepy all day, unable to sleep, daytime sleepiness, Do I have a sleep disorder, Sleep disorder home test , Signs of sleep disorder , Test for sleep disorder, Symptoms of sleep disorder, Fatigue , Lack of energy, Burned out, Tired
