# Supplementary material for: Identifying Sleep Disorders From Search Engine Activity: Combining User-Generated Data With a Clinically Validated Questionnaire
Source: J Med Internet Res. 2022 Nov 23;24(11):e41288. doi: 10.2196/41288 (PMC9730212; doi:10.2196/41288)
Supplement: Multimedia Appendix 2 [file jmir_v24i11e41288_app2.docx]

## Appendix B: Campaign statistics

Figure S1 shows the number of impressions per day, the clickthrough rate and the conversion rate over the duration of the campaign. As the figure shows, the number of impressions was high initially, but then dropped down to around 2000 impressions per day, even though the campaign budget remained the same. On the other hand, clickthrough rate and conversion rate rose over time, indicating that the advertising campaign learned to focus on people who were more likely to click and complete the DSQ.

| 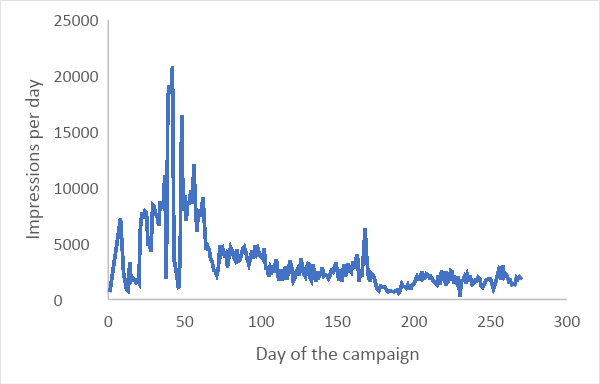 |
| --- |
| 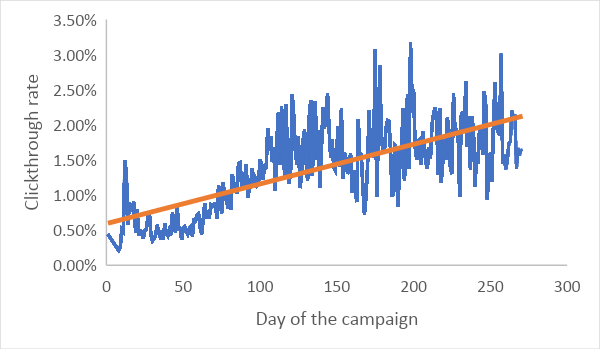 |
| 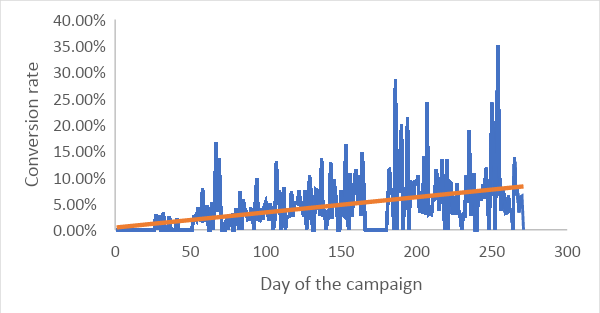 |

*Figure S1: Impressions per day (top), clickthrough rate (middle) and conversion rate (bottom) over the duration of the campaign. The bold lines in the second and third charts are a 7-day moving average.*

The keywords with the highest CTR and the highest conversion rate are shown in Table S1. Spearman correlation between CTR and conversion rate, for keywords with at least 100 impressions, was 0.23 (P=0.04). Similarly, the ads with the highest CTR and the highest conversion rate are shown in Table S2. Spearman correlation between CTR and conversion rate was 0.50 (P=0.10). Thus, the correlation between CTR and conversion rate is not statistically significant (after applying Bonferroni correction).

| Highest CTR | Do I have a delayed sleep phase syndrome  Test for insufficient sleep syndrome  Pauses in breathing during sleep  Can't wake up in the morning  Pauses in breathing at night  Do I have a insufficient sleep syndrome  Delayed sleep phase syndrome home test  Symptoms of delayed sleep phase syndrome  Test for insomnia  Causes of daytime sleepiness |
| --- | --- |
| Highest conversion rate | How to treat suspected sleep apneas  Suspected sleep apnea  Sleeping problems causes  How to get quality sleep  Sleep disorder home test  Lack of energy  Symptoms of delayed sleep phase syndrome  Can't wake up in the morning  Feeling sleepy all day  Do I have a insomnia |

*Table S1: Keywords with the highest CTR and highest conversion rate*

| Highest CTR | How well do you sleep? Answer a sleep assessment questionnaire and get free sleep training plan  Are you feeling tired? Take our clinically validated sleep assessment. It takes only a few minutes.  Sleep difficulties? Take a clinically validated sleep assessment and get your free sleep app!  Worried about your sleep? Take a free short sleep assessment now! Get a personal sleep report and a free sleep app.  Your sleep is important. It’s never too late! Take our clinically validated sleep assessment now - free of charge  No sleep? There's a solution. Tired of feeling tired? Download dayzz, your free pocket-sized sleep trainer.  Better sleep is possible. Download dayzz app, get your sleep assessment and start improving your sleep  Get your free sleep assessment. Download dayzz app, get your sleep assessment and start improving your sleep  How is your sleep? Take our clinically validated sleep assessment and discover how you really sleep  Can't sleep? Sleepless nights have a solution – get a free personalized sleep training plan  Get your free sleep assessment. Sleepless nights have a solution – get a free personalized sleep training plan  Can’t sleep? Sleepless nights have a solution – get a free personalized sleep training plan |
| --- | --- |
| Highest conversion rate | Get your free sleep assessment. Download dayzz app, get your sleep assessment and start improving your sleep  How well do you sleep? Answer a sleep assessment questionnaire and get free sleep training plan  Are you feeling tired? Take our clinically validated sleep assessment. It takes only a few minutes.  Sleep difficulties? Take a clinically validated sleep assessment and get your free sleep app!  How is your sleep? Take our clinically validated sleep assessment and discover how you really sleep  Get your free sleep assessment. Sleepless nights have a solution – get a free personalized sleep training plan  No sleep? There's a solution. Tired of feeling tired? Download dayzz, your free pocket-sized sleep trainer.  Your sleep is important. It’s never too late! Take our clinically validated sleep assessment now - free of charge  Worried about your sleep? Take a free short sleep assessment now! Get a personal sleep report and a free sleep app.  Better sleep is possible. Download dayzz app, get your sleep assessment and start improving your sleep  Can’t sleep? Sleepless nights have a solution – get a free personalized sleep training plan  Can't sleep? Sleepless nights have a solution – get a free personalized sleep training plan |

*Table S2: Ads with the highest CTR and highest conversion rate*
